# Metabolic syndrome in patients with prostate cancer

**DOI:** 10.1590/S1516-31802008000500006

**Published:** 2008-09-04

**Authors:** Iúri Amorim de Santana, Gustavo Souza Moura, Nivaldo Farias Vieira, Rosana Cipolotti

**Keywords:** Prostate cancer, Prostate-specific antigen, Metabolic syndrome X, Obesity, Hyperglycemia, Câncer de próstata, Antígeno prostático específico, Síndrome X metabólica, Obesidade, Hiperglicemia

## Abstract

**CONTEXT AND OBJECTIVE:**

Prostate cancer (PCa) is the second most common cancer among men in Brazil. Recently, several studies have hypothesized a relationship between PCa and metabolic syndrome (MS). The aim here was to identify an association between MS and PCa.

**DESIGN AND SETTING:**

Cross-sectional study, Fundação de Beneficência Hospital de Cirurgia (FBHC) and Universidade Federal de Sergipe.

**METHODS:**

Laboratory and anthropometric parameters were compared between PCa patients (n = 16) and controls (n = 16).

**RESULTS:**

The PCa patients showed significantly greater frequency of MS than did the controls (p = 0.034). Serum glucose was higher and high-density lipoprotein-cholesterol was lower than in the controls, although without significant differences. There were significant differences in blood pressure (p = 0.029) and waist-to-hip ratio (p = 0.004). Pearson linear correlation showed a positive association between waist-to-hip ratio and prostate specific antigen (r = 0.584 and p = 0.028). Comparing subgroups with and without MS among the PCa patients, significant differences (p < 0.05) in weight, height, body mass index, hip circumference and lean body mass were observed, thus showing higher central obesity in those with MS. The serum glucose values were also higher in MS patients (p = 0.006), thus demonstrating that insulin resistance has a role in MS physiopathology.

**CONCLUSIONS:**

Our study suggests that MS may exert an influence on the development of PCa. However, it would be necessary to expand the investigation field with larger sample sizes and cohorts studied, to test the hypothesis generated in this study.

## RESUMO

**CONTEXTO E OBJETIVO:**

O câncer de próstata (CaP) é o segundo câncer mais comum entre os homens no Brasil. Recentemente diversos estudos têm apresentado a hipótese de que o CaP possa estar relacionado à síndrome metabólica (SM). O objetivo é identificar associação entre SM e CaP.

**IPO DE ESTUDO E LOCAL:**

Estudo transversal analítico. Fundação de Beneficência Hospital de Cirurgia (FBHC) e Universidade Federal de Sergipe.

**MÉTODOS:**

Foram comparados parâmetros laboratoriais e antropométricos entre um grupo com CaP (n = 16) e um grupo controle (n = 16).

**RESULTADOS:**

O grupo com CaP apresentou ocorrência significativamente maior de SM em relação ao grupo controle (p = 0,034). A glicemia foi superior e a lipoproteína de alta densidade-colesterol (HDL-c) inferior quando comparados ao controle, contudo sem diferença significativa. Foi observada diferença significativa quanto à pressão arterial (p = 0,029) e à relação cintura/quadril (p = 0,004). A correlação linear de Pearson revelou associação positiva significativa entre o valor da relação cintura/quadril e o nível de antígeno prostático específico (r = 0,584 e p = 0,028). Comparando-se os subgrupos com SM e sem SM dentro do grupo com CaP, houve diferenças significativas (p < 0,05) quanto a peso, altura, índice de massa corporal, circunferência do quadril e massa magra, evidenciando o maior grau de obesidade do tipo central nos indivíduos com SM. Os níveis glicêmicos foram também significativamente maiores naqueles com SM (p = 0,006), demonstrando o papel da resistência insulínica na fisiopatologia da SM.

**CONCLUSÕES:**

Nosso estudo sugere que a SM exerce influência sobre o desenvolvimento do CaP, contudo é necessário ampliar o campo de investigação, com estudos com maior número de indivíduos, e avaliando também os hábitos e estilo de vida.

## INTRODUCTION

Prostate cancer (PCa) is the second most common cancer among men in Brazil, overtaken only by non-melanoma skin cancer. In 2006, Brazil had an incidence of 51.41 cases per 100,000 inhabitants, with 47,280 new cases. In the State of Sergipe in the same year, 350 new cases were registered (among which 170 were in the capital city, Aracaju), corresponding to 36.14 cases per 100,000 inhabitants.^1^

Recently, the hypothesis of a relationship between PCa and a group of metabolic abnormalities known as metabolic syndrome (MS) has been put forward.^2^ This syndrome has been defined in different ways, but its physiopathology is still not well known. It is characterized by central obesity, insulin resistance, high serum glucose levels, systemic arterial hypertension and dyslipidemia.^3^

The prevalence of MS has been increasing worldwide,^4^ and it has become a major public health problem in several countries. In Brazil, epidemiological reports also show this increase,^5^ and in the United States, which has one of the highest prevalences, it may reach 40% of the population.^6^

Among the physiopathological entities that comprise MS, the serum level of insulin-like growth factor I (IGF-I) seems to be the one that is most closely linked with PCa.^7^ The hyperinsulinemia observed in patients with MS is the factor responsible for stimulating the production of IGF-I in the liver. This, as a potent mitogenic factor and apoptosis inhibitor, increases the cell turnover, which results in higher susceptibility to malignant transformation of cells. Serum levels of IGF-I higher than 60 ng/ml seem to be associated with an increased risk of development of PCa.^8^

Another metabolic pathway possibly involved consists of increase in cholesterol levels. Cholesterol is an essential constituent of the cell plasmatic membrane, but when its levels become higher than a critical concentration, it can inhibit cell apoptosis through structures known as lipids rafts, which alter the mechanisms of signal transduction.^9^

Beyond the possibility that MS may play a role in the development of PCa, some studies have shown that after this disease has become established, MS may worse its prognosis, through accelerating its progression. Other studies evaluating the relationship between MS and PCa have shown conflicting results.^10-15^

## OBJECTIVE

The present study aimed to evaluate the association between MS and PCa.

## METHODS

This was a controlled cross-sectional study with groups composed by men aged between 59 and 82 years. The cases included 16 men with a histological diagnosis of prostate adenocarcinoma who were being followed up at the oncology center of Fundação de Beneficência Hospital de Cirurgia (FBHC) in the city of Aracaju, Sergipe, Brazil. The sample size was determinate by convenience, limited by the number of patients in the oncology center. Patients with Karnofsky performance status (KPS)^16^ lower than 70%, disseminated disease and chronic use of systemic corticosteroids were excluded. Sixteen age-matched men without any history of benign or malignant prostate disease who were living in Aracaju and had the same social background were selected for the control group.

The evaluations included obtaining the patients' clinical histories, with any pathological and familial background of systemic arterial hypertension, cardiovascular disease, diabetes mellitus, dyslipidemia or cancer. All clinical evaluations were performed in FBHC. Anthropometric measurements were made, including weight, height and waist and hip circumferences. The diagnosis criteria for MS were defined in accordance with the recommendations of the International Diabetes Federation (IDF).^17^ These included waist circumference greater than the reference values, and at least two other criteria: (1) fasting glucose ≥ 5.6 mmol/l (100 mg/dl) or previously diagnosed type 2 diabetes; (2) triglycerides ≥ 1.7 mmol/l (150 mg/dl) and/or specific treatment for this lipid abnormality; (3) high-density lipoprotein-cholesterol (HDL-c) level < 1.0 mmol/l (40 mg/dl) in men or < 1.3 mmol/l (50 mg/dl) in women; and (4) arterial hypertension: blood pressure ≥ 130 x 85 mmHg and/or treatment of previously diagnosed hypertension. The waist circumference was measured at the midline between the lower costal margin and the iliac crest, and the hip circumference was measured at the level of the greater trochanter of the femur. A flexible unstretchable measuring tape with an accuracy of 0.1 cm was used to measure waist and hip circumferences.

Based on these results, the following were calculated:

(1) body mass index (BMI) [weight (kg) ÷ height (m)^2^];
(2) waist-to-hip ratio [waist (cm) ÷ hip circumference (cm)]; and
(3) lean body mass (LBM) [2.447 - 0.09516 age (years) + 0.1074 height (cm) + 0.3362 weight (kg) ÷ 0.732].

Blood pressure was measured in accordance with the guidelines of the Brazilian Society of Arterial Hypertension,^18^ and aneroid sphygmomanometers were used within the standards established by the National Institute of Metrology, Standardization and Industrial Quality (Inmetro). The tumors were classified by their histological grade in accordance with the Gleason scale.^19^

Blood collection for the biochemical tests [total cholesterol, HDL-c, low-density lipoprotein-cholesterol (LDL-c) and fasting glucose] was performed after 12 hours of fasting. The samples were processed at the laboratory of Universidade Federal de Sergipe, in accordance with the methods standardized by the Brazilian Committee of Clinical Analysis and *in vitro* Diagnosis.^20^

The data were analyzed using the Statistical Package for the Social Science (SPSS 13.0^®^). Quantitative variables were compared using Student's t test and the qualitative variables using Fisher's Exact Test. The power of association between the quantitative variables was determined using Pearson's linear correlation. p-values < 0.05 were taken to be significant.

This study was conducted with the approval of the Research Ethics Committee of Universidade Federal de Sergipe.

## RESULTS

In this study, two groups of similar ages originating from the same social background were evaluated. The mean age of the cases was 67 ± 6.57 years and the mean age of the controls was 65.06 ± 5.47 years old (p = 0.372).

The cases presented significantly greater occurrence of MS than did the control group (p = 0.034; Table 1). The case group had more hypertension that had previously been diagnosed (p = 0.029) and higher levels of arterial blood pressure at the time of the interview. There was also a tendency towards higher frequency of diabetes mellitus type 2 in the case group, but this did not reach statistical significance (p = 0.172) Table 1.

**Table 1.** Epidemiological profile of the case group (prostatic cancer) and control group

	Cases	Controls	p
Metabolic syndrome	11 (68.7%)	4 (25.0%)	0.034*
Diabetes mellitus type 2	5 (15.6%)	1 (3.12%)	0.172
Dyslipidemia	4 (12.5%)	7 (21.8%)	0.458
Systemic arterial hypertension	13 (40.62%)	6 (18.7%)	0.029*
Family history of cancer	4 (12.5%)	7 (21.85%)	0.458
Family history of cardiovascular disease	6 (18.7%)	4 (12.5%)	0.704

* statistical significance (p < 0.05).

Table 2 summarizes the results regarding the clinical and laboratorial characteristics of the case and control groups. The blood pressure was significantly higher in the case group and there was a tendency towards higher blood glucose values and lower levels of HDL-c, although without statistical significance. The patients with PCa also showed significantly higher waist-to-hip ratios [1.03 (± 0.04) versus 0.98 (± 0.05); p = 0.004], lower height [1.61 (± 0.07) versus 1.68 (± 0.07); p = 0.017] and lower serum level of total cholesterol [181.25 (± 36.17) versus 208.50 (± 38.46); p = 0.048]. The control group had greater waist circumference and higher triglyceride levels, although these differences did not reach statistical significance.

**Table 2.** Comparison of means of clinical and laboratory variables between the case group (prostatic cancer) and control group

	Cases Mean (± SD)	Controls Mean (± SD)	p
Age (years)	67.00 (± 6.57)	65.06 (± 5.47)	0.372
Weight (kg)	71.56 (± 11.17)	73.06 (± 11.12)	0.706
Height (m)	1.61 (± 0.07)	1.68 (± 0.07)	0.017*
Body mass index (kg/m^2^)	27.30 (± 3.76)	25.73 (± 3.12)	0.212
Hip circumference (cm)	93.25 (± 9.11)	99.31 (± 9.09)	0.069
Waist circumference (cm)	97.18 (± 11.23)	98.62 (± 11.15)	0.719
Waist-to-hip ratio	1.03 (± 0.04)	0.98 (± 0.05)	0.004*
Systolic pressure (mmHg)	157 (± 17.84)	136.88 (± 18.51)	0.004*
Diastolic pressure (mmHg)	91.50 (± 9.94)	83.13 (± 8.73)	0.017*
Fasting blood glucose (mg/dl)	101.6 (± 30.16)	92.81 (± 13.97)	0.332
Total cholesterol (mg/dl)	181.25 (± 36.17)	208.50 (± 38.46)	0.048*
LDL-c (mg/dl)	115.93 (± 31.30)	120.93 (± 42.35)	0.716
HDL-c (mg/dl)	36.30 (± 10.75)	41.69 (± 9.58)	0.145
Triglycerides (mg/dl)	148.50 (± 111.90)	204.50 (± 116.43)	0.103
Lean body mass (%)	65.01 (± 2.38)	65.83 (± 2.21)	0.324

SD = standard deviation; LDL-c = low-density lipoprotein-cholesterol; HDL-c = high-density lipoprotein-cholesterol.

* statistically significant (p < 0.05).

Subgroups of patients with and without MS were analyzed within the case group. The data are shown in Tables 3 and 4 and present significant differences in weight, height, BMI, waist circumference and LBM. This result highlights the higher level of central obesity in the subgroup with MS. It is worth noting that the blood glucose levels were significantly higher in the cases with MS, thus reinforcing the notion that insulin resistance has a role in the physiopathology of this syndrome Table 4.

**Table 3.** Epidemiological profile of subgroups with and without metabolic syndrome in the prostate cancer group

	With metabolic syndrome n (%)	Without metabolic syndrome n (%)	p
Diabetes mellitus type 2	3 (33.3%)	2 (28.6%)	1.000
Dyslipidemia	3 (33.3%)	1 (14.3%)	0.585
Systemic arterial hypertension	6 (66.7%)	6 (85.7%)	0.585
Family history of cancer	2 (22.2%)	2 (28.6%)	1.000
Family history of cardiovascular disease	4 (44.4%)	2 (28.6%)	0.633
Gleason score > 6	2 (14.3%)	2 (14.3%)	0.580

**Table 4.** Comparison of means of clinical and laboratory variables between subgroups with and without metabolic syndrome

	With metabolic syndrome Mean (± SD)	Without metabolic syndrome Mean (± SD)	p
Age (years)	65.11 (± 5.18)	69.43 (± 7.74)	0.142
Weight (kg)	75.7 (± 4.60)	66.24 (± 15.00)	0.001*
Height (m)	1.60 (± 0.46)	1.62 (± 0.10)	0.048*
Body mass index (kg/m^2^)	29.178 (± 1.87)	24.88 (± 4.32)	0.037*
Hip circumference (cm)	96.55 (± 3.84)	85.14 (± 12.40)	0.018*
Waist circumference (cm)	102.55 (± 5.02)	85.85 (± 12.50)	0.056
Waist-to-hip ratio	1.05 (± 0.03)	1.00 (± 0.02)	0.148
Systolic pressure (mmHg)	163.11 (± 18.22)	149.14 (±15.00)	0.530
Diastolic pressure (mmHg)	95.11 (± 11.79)	86.86 (± 4.14)	0.084
Fasting blood glucose (mg/dl)	114.67 (± 34.49)	83.57 (± 7.36)	0.006*
Total cholesterol (mg/dl)	190.44 (± 41.48)	169.71 (± 26.45)	0.465
LDL-c (mg/dl)	121.60 (± 35.84)	109.45 (± 26.36)	0.953
HDL-c (mg/dl)	32.09 (± 9.80)	42.00 (± 9.89)	0.845
Triglycerides (mg/dl)	193.22 (± 133.66)	91.00 (± 25.04)	0.103
Lean body mass (%)	63.85 (± 1.09)	66.5 (± 2.82)	0.004*
Prostatic specific antigen	35.7 (± 56.53)	17.08 (± 7.58)	0.188

SD = standard deviation; LDL-c = low-density lipoprotein-cholesterol; HDL-c = high-density lipoprotein-cholesterol.

* statistically significant (p < 0.05).

The presence of MS did not modify the histological grade of the tumor at the time of diagnosis, since both subgroups had similar frequencies of high-grade tumors (Gleason > 6; Table 4).

There was a positive linear correlation between the waist-to-hip ratio and serum level of prostate-specific antigen (PSA) at the time of diagnosis (r = 0.584 and p = 0.028; Table 5).

**Table 5.** Linear correlation between prostate-specific antigen and anthropometric markers

	Prostate specific antigen	
	Correlation coefficient (r)	p
Weight (kg)	0.008	0.979
Height (m)	0.137	0.642
Body mass index (kg/m^2^)	- 0.088	0.765
Waist circumference (cm)	0.286	0.322
Hip circumference (cm)	0.128	0.662
Waist-to-hip ratio	0.553	0.040*

* statistical significance (p < 0.05).

## DISCUSSION

The results showed that there was significantly greater occurrence of MS in the PCa group, thus corroborating the hypothesis that MS may have some influence on the development of PCa, or even lead to a worse outcome.

Since three out of the five criteria are needed for a diagnosis of MS, the combinations found in the present study gave rise to significant occurrence of MS among the PCa patients, although differences between the case and control groups were found only for blood pressure, which was higher in the case group. The combination of variables for diagnosing MS explains the higher occurrence of this disease in the case group, even though the mean values of each variable individually were not significantly different.

The components of MS may be considered to be markers relating to an underlying metabolic defect that, in turn, via increased insulin and IGF-I levels, might promote the development of clinical PCa. IGF-I can mimic the effects of steroid hormones, thereby promoting prostate cell growth and increased mitosis rate, or even inhibiting apoptosis, which is an important mechanism in the carcinogenesis process. A previous case-control study^7^ showed significantly higher levels of IGF-I in the PCa group (p = 0.006). There was also a strong dose-dependent association between increasing IGF-I plasma levels and PCa risk (odds ratio, OR = 3.92; 95% confidence interval, CI = 1.58 - 9.70), when IGF-I levels were higher than 151.7 ng/ml. In the present study, hyperglycemia was considered to be an indirect marker for hyperinsulinemia. Significantly higher fasting blood glucose levels were observed in MS patients. Likewise, the PCa group also presented higher levels of blood glucose, although without reaching statistical significance. A cohort study^21^ confirmed that serum glucose is a positive risk factor for PCa (relative risk, RR = 1.11; 95% CI = 1.01-1.22).

The waist-to-hip ratio is a trustworthy indirect parameter for measuring central obesity, which is fundamental for determining MS physiopathology and is included in the World Health Organization (WHO) criteria.^22^ In our sample, significantly higher values of waist-to-hip ratio in the case group were observed. Furthermore, there was a positive relationship between central obesity and PSA at diagnosis. The waist-to-hip ratio had an RR for PCa of 1.11 per 0.1 unit increment, (95% CI = 0.95-1.30) in a previous study.^15^ These results support the proposed hypothesis that visceral fat has a role in MS physiopathology and has a direct influence promoting PCa.

Several studies have evaluated the correlation between anthropometric characteristics and PCa development. An association between BMI and PCa has already been demonstrated in several recent studies.^23-27^ Some of these studies have shown that BMI is related to higher mortality among PCa patients and that there may be a positive relationship between BMI and disease stage.^26,27^ A recent meta-analysis reviewed 55,521 cases of PCa that were identified among 2,818,767 men in 31 cohort studies, along with 13,232 cases and 16,317 controls from 25 case-control studies. The risk ratio (RR) of developing PCa was 1.05 per 5 kg/m^2^ increase in BMI (95% CI 1.01-1.08). The results stratified by disease stage showed that the association with BMI was greater (p = 0.02) for the risk of advanced disease (RR 1.12 per 5 kg/m^2^ increase; 95% CI: 1.01-1.23) than for the risk of localized disease (RR 0.96 per 5 kg/m^2^ increase; 95% CI: 0.89-1.03).^15^ Severson et al.^28^ observed a positive association between BMI and the incidence of PCa, and suggested that the LBM rather than the body fat played a part in the development of PCa. Here, the group of PCa patients had higher BMI and lower LBM, although these results were not statistically significant. BMI is an imperfect measure of obesity that combines adipose and non-adipose body components.^29^ Thus, the positive association between BMI and PCa can be related to the percentage of muscle mass, which possibly represents a higher level of androgens in the organism. LBM is considered important in androgen-dependent diseases such as PCa, but the calculated LBM index may not be correct because the formula assumes that the proportion of water in the LBM is constant. A study using dual X-ray absorptiometry and bioelectric impedance analysis techniques (gold standard) to evaluate body composition among non-metastatic PCa patients showed significantly lower LBM than in a control group.^30^

In the present study, the total cholesterol level was significantly higher in the control group. This hypocholesterolemia may, in fact, be the result rather than the cause of PCa, since it has already been demonstrated that the presence of cancer can cause decreased levels of this substance.^31^ This has been confirmed by several epidemiological studies that demonstrated that people with cancer present lower levels of cholesterol, for reasons that are still unknown.^31,32^ However, the role of cholesterol in the physiopathology of PCa is still controversial. Studies have provided evidence that cholesterol is a mediator of signal transduction processes that are important for PCa tumor cell survival and disease progression.^9^ Cholesterol is a precursor of androgenic hormones, which interfere directly in the process of genesis and progression of PCa.^10^

Severson et al.^33^ attributed the relationship that we also found between PCa and MS to the patients' lifestyles, including their eating habits and physical activity. Some studies have linked diet and cancer. There are hypotheses about the protective role of soybeans, which are rich in isoflavones, substances that are also known as phytoestrogens.^34,35^

Significant quantities of data on the role of physical activity in primary prevention against hormone-dependent cancers like colon, endometrium, breast and prostate cancers have been accumulated,^36-38^ but the physiological pathways underlying this relationship are not well understood.^37^ Physical activity modulates blood testosterone levels by decreasing the production of this hormone as well as increasing the levels of binding proteins for sexual hormones. This causes decreases in the free fraction of the hormone, which in turn is responsible for the effect of this hormone on target tissues.^37^ Another mechanism could involve reductions in insulin and IGF-I synthesis. Nevertheless, the most widely accepted hypothesis for the pathway is that the physical activity, in conjunction with decreased calorie intake and decreased body weight, increases the production of insulin-like growth factor binding proteins (IGFBP-3), thereby decreasing the action of IGF-I.^37^

## CONCLUSION

Our study suggests that MS may exert an influence on the development of PCa. However, it would be necessary to expand the investigation field with larger sample sizes and cohort studies, to test the hypothesis generated in this study.

## References

[1] Brasil, Ministério da Saúde (2002). Secretaria Nacional de Assistência à Saúde. Instituto Nacional de Câncer. Coordenação de Prevenção e Vigilância — Conprev Câncer da Próstata: consenso.

[2] Fowke JH, Signorello LB, Chang SS (2006). Effects of obesity and height on prostate-specific antigen (PSA) and percentage of free PSA levels among African-American and Caucasian men. Cancer.

[B3] Sociedade Brasileira de Hipertensão (2004). Conceituação, epidemiologia e diagnóstico. Revista da Sociedade Brasileira de Hipertensão.

[4] Hsing AW, Tsao L, Devesa SS (2000). International trends and patterns of prostate cancer incidence and mortality. Int J Cancer.

[5] Oliveira EP, Souza MLA, Lima MDA (2006). Prevalência de síndrome metabólica em uma área rural do semi-árido baiano. [Prevalence of metabolic syndrome in a semi-arid rural area in Bahia]. Arq Bras Endocrinol Metab.

[6] Ford ES (2005). Prevalence of the metabolic syndrome defined by the International Diabetes Federation among adults in the U.S. Diabetes Care.

[7] Hammarsten J, Högstedt B. (2005). Hyperinsulinaemia: a prospective risk factor for lethal clinical prostate cancer. Eur J Cancer.

[8] Gomella LG, Labrie F, Gamito EJ, Brawer MK (2001). Prostate cancer: risk assessment and diagnostic approaches. Rev Urol.

[9] Zhuang L, Kim J, Adam RM, Solomon KR, Freeman MR (2005). Cholesterol targeting alters lipid raft composition and cell survival in prostate cancer cells and xenografts. J Clin Invest.

[10] Mydlo JH, Tieng NL, Volpe MA, Chaiken R, Kral JG (2001). A pilot study analyzing PSA, serum testosterone, lipid profile, body mass index and race in a small sample of patients with and without carcinoma of the prostate. Prostate Cancer Prostatic Dis.

[11] Thompson MM, Garland C, Barrett-Connor E, Khaw KT, Friedlander NJ, Wingard DL (1989). Heart disease risk factors, diabetes, and prostatic cancer in an adult community. Am J Epidemiol.

[12] Giovannucci E, Rimm EB, Stampfer MJ, Colditz GA, Willett WC (1997). Height, body weight, and risk of prostate cancer. Cancer Epidemiol Biomarkers Prev.

[13] Sox HC (1999). Current controversies in screening: cholesterol, breast cancer, and prostate cancer. Mt Sinai J Med.

[14] Schuurman AG, Goldbohm RA, Dorant E, van den Brandt PA (2000). Anthropometry in relation to prostate cancer risk in the Netherlands Cohort Study. Am J Epidemiol.

[15] MacInnis RJ, English DR (2006). Body size and composition and prostate cancer risk: systematic review and meta-regression analysis. Cancer Causes Control.

[16] Schag CC, Heinrich RL, Ganz PA (1984). Karnofsky performance status revisited: reliability, validity, and guidelines. J Clin Oncol.

[B17] International Diabetes Federation (2005). The IDF consensus worldwide definition of the metabolic syndrome.

[18] Sociedade Brasileira de Hipertensão (2006). V Diretrizes Brasileiras de Hipertensão Arterial. Hipertensão.

[19] Gleason DF (1966). Classification of prostatic carcinomas. Cancer Chemother Rep.

[B20] Sociedade Brasileira de Análises Clínicas (1998). Secretarias Técnicas. Comitê Brasileiro de Análises Clínicas e Diagnóstico in vitro. http://www.sbac.org.br/pt/conteudos/secretarias_tecnicas/abnt_cb_36.htm#2.

[21] Tulinius H, Sigfússon N, Sigvaldason H, Bjarnadóttir K, Tryggvadóttir L. (1997). Risk factors for malignant diseases: a cohort study on a population of 22,946 Icelanders. Cancer Epidemiol Biomarkers Prev.

[B22] World Health Organization (1999). Definition, diagnosis and classification of diabetes mellitus and its complications: report of a WHO consultation. Part 1: diagnosis and classification of diabetes mellitus. World Health Organization.

[23] Calle EE, Rodriguez C, Walker-Thurmond K, Thun MJ (2003). Overweight, obesity, and mortality from cancer in a prospectively studied cohort of U.S. adults. N Engl J Med.

[24] Veierod MB, Laake P, Thelle DS (1997). Dietary fat intake and risk of prostate cancer: a prospective study of 25,708 Norwegian men. Int J Cancer.

[25] Irani J, Lefebvre O, Murat F, Dahmani L, Doré B. (2003). Obesity in relation to prostate cancer risk: comparison with a population having benign prostatic hyperplasia. BJU Int.

[26] Fitzpatrick AL, Daling JR, Furberg CD, Kronmal RA, Weissfeld JL (2001). Hypertension, heart rate, use of antihypertensives, and incident prostate cancer. Ann Epidemiol.

[27] Engeland A, Tretli S, Bjorge T. (2003). Height, body mass index, and prostate cancer: a follow-up of 950000 Norwegian men. Br J Cancer.

[28] Severson RK, Grove JS, Nomura AM, Stemmermann GN (1988). Body mass and prostatic cancer: a prospective study. BMJ.

[29] MacInnis RJ, English DR, Gertig DM, Hopper JL, Giles GG (2003). Body size and composition and prostate cancer risk. Cancer Epidemiol Biomarkers Prev.

[30] Maturo G, Vespasiani G, Mohamed EI (2003). Evaluating body composition of Italian prostate cancer patients without metastases. Acta Diabetol.

[31] Alexopoulos CG, Blatsios B, Avgerinos A. (1987). Serum lipids and lipoprotein disorders in cancer patients. Cancer.

[32] Fiorenza AM, Branchi A, Sommariva D. (2000). Serum lipoprotein profile in patients with cancer. A comparison with non-cancer subjects. Int J Clin Lab Res.

[33] Severson RK, Nomura AM, Grove JS, Stemmermann GN (1989). A prospective study of demographics, diet, and prostate cancer among men of Japanese ancestry in Hawaii. Cancer Res.

[34] Fournier DB, Erdman JW, Gordon GB (1998). Soy, its components, and cancer prevention: a review of the in vitro, animal, and human data. Cancer Epidemiol Biomarkers Prev.

[35] Lee MM, Gomez SL, Chang JS, Wey M, Wang RT, Hsing AW (2003). Soy and isoflavone consumption in relation to prostate cancer risk in China. Cancer Epidemiol Biomarkers Prev.

[36] Lee IM Sesso HD Chen JJPaffenbarger RS Jr. Does physical activity play a role in the prevention of prostate cancer? Epidemiol Rev 2001 23 1 132 137 11588837 10.1093/oxfordjournals.epirev.a000778

[37] Friedenreich CM, Orenstein MR (2002). Physical activity and cancer prevention: etiologic evidence and biological mechanisms. J Nutr.

[38] Friedenreich CM, McGregor SE, Courneya KS, Angyalfi SJ, Elliott FG (2004). Case-control study of lifetime total physical activity and prostate cancer risk. Am J Epidemiol.

